# Epidemiology of primary brain tumors in childhood and adolescence in Kuwait

**DOI:** 10.1186/2193-1801-2-58

**Published:** 2013-02-18

**Authors:** Kenneth Chukwuka Katchy, Susan Alexander, Nabila Mohammed Al-Nashmi, Abbas Al-Ramadan

**Affiliations:** 1 Department of Pathology, Al-Sabah Hospital, Safat, Kuwait; 2 Department of Neurosurgery, Ibn Sina Hospital, Safat, Kuwait; 3 FRCPath, FRCPC, Department of Pathology, Al-Sabah Hospital, P.O.Box 4078, 13041 Safat, Kuwait

**Keywords:** Epidemiology, Primary brain tumors, Childhood, Adolescence, Kuwait

## Abstract

The relatively high frequency of primary brain tumors (PBT) observed in childhood and adolescence in Kuwait has necessitated this epidemiological study. It is based on the records of the Department of Pathology, Al-Sabah Hospital, which examined all brain tumor biopsies done in this age group in Kuwait between 1995 and 2011. During this period, 75 boys (49%) boys and 77 (51%) girls had histologically confirmed PBT. They comprised 122 children (0–14 years) and 30 adolescents (15–19 years). The boys/girls ratio was 1.03 in childhood and 0.76 in adolescence. The age-adjusted incidence rate was 11.2/ million person-years. Early childhood (0–4 years) had the peak frequency of tumors (33%), highest adjusted age-specific incidence rate (3.8/million person-years) of all tumors and the least boys/girls rates ratio (0.38) for astrocytic tumors. Low grade and high grade tumors peaked in 5–9 and 0–4 years respectively. Risk factors (hereditary syndromes or previous radio-therapy) were identified in three patients. Three (2%) tumors were congenital. High grade tumors comprised 47% of childhood and 23% of adolescence PBT. The most common tumors in childhood were astrocytoma (37%), embryonal tumors (31%), ependymoma (8%), and in adolescence astrocytoma (27%), pituitary adenoma (23%) and glioblastoma (13%). Embryonal tumors formed 44% of PBT in early childhood. Gliomas constituted 54% and 43% of all PBT, but 25% and 57% of high grade tumors in childhood and adolescence respectively. Most common tumor locations were cerebellum (47%), ventricles (19%) and cerebral lobes (17%) in childhood and pituitary (30%), cerebellum (27%) and 13% each for cerebral lobes and ventricles in adolescence. Approximately 57% of childhood and 23% of adolescence PBT were infratentorial.

In conclusion, despite the high relative frequency of PBT before the age of 20 years in Kuwait, its incidence rate is apparently low. Compared with Western countries, Kuwait has a lower incidence of malignant gliomas, but a higher frequency of cerebellar and intraventricular tumors. Embryonal tumors are remarkably common in early childhood.

## Introduction

Primary brain tumors (PBT) are a varied group of benign and malignant tumors which arise from the brain parenchyma and its surrounding structures. Although an overwhelming majority of these tumors occur in adults, the pediatric and adolescent PBT deserve special attention for several reasons. Firstly, some specific histological types which are common in childhood are rare in adults. More importantly there are indications that pediatric and adult glial tumors have significant differences in their molecular biology and behavior. These have serious implications for future research, treatment and prognostic factors (Pediatric Brain Tumors 2005). Secondly, there is a relatively high probability of delayed diagnosis in adolescence because symptomatic cases may be either misconstrued as just adolescence behavior or attributed to hormonal changes that occur during this period (Kieran et al. 2010). Thirdly, compared to adult tumors, a disproportionately higher percentage of childhood and adolescence PBT are malignant. Indeed, in many parts of the world, they are not only the most common malignant solid tumors seen before the age of 20 years, but also “the greatest cause of childhood cancer mortality in the age group 0–14” (El basmi & Al-Asfour 2007; IBTA/UICC 2009; Miltenburg et al. 1996). In recent times, early detection and improvement in therapeutic modalities have resulted in longer survival. Unfortunately, these therapeutic modalities by themselves have deleterious effects on the brain tissue. Consequently, longer survivals are often associated with neurological, cognitive and endocrine disorders as well as decreased quality of life (Serafim et al. 2001). Besides, some survivors have an increased risk of developing a second neoplasm later in life (Broniscer et al. 2004). Therefore, PBT in childhood and adolescence constitute a tremendous burden for families and health care delivery systems and have been aptly described by Kun as “perhaps the most vexing area of pediatric oncology” ( 2009).

As with most tumors, the etiology of PBT is unknown. Mueller and Gurney have suggested that their etiology may be multi-factorial and includes both genetic and environmental factors (Mueller & Gurney 2005). Ionizing radiation (Ohgaki & Kleihues 2005) and some hereditary disorders are apparently the only accepted risk factors for childhood PBT. These genetic syndromes account for only few cases (< 5%) of PBT (Bondy et al. 2008; Narod et al. 1991). Attempts to link childhood PBT to pre- and peri-natal exposures to obnoxious environmental agents have produced interesting, but at times, inconclusive or inconsistent results (Baldwin & Preston-Martin 2004; Schüz et al. 2001; Clapp et al. 2008; Efird et al. 2005).

Reports in literature suggest that there are world-wide variations in the pattern of childhood and adolescence PBT with respect to incidence rates, gender preference, anatomical location and relative frequency of specific histological types. Location and histological types may, to a large extent, influence treatment modalities, outcome and search for risk factors. Data on both parameters are considered useful for planning of health care delivery system and future research in any given locality.

The observation that Kuwait has an inexplicably high proportion (16%) of PBT in childhood and adolescence (Katchy et al. 2011) has necessitated the need for a more detailed examination of PBT in this group in Kuwait. The objective of this study is epidemiological and represents the first attempt at estimating incidence rates of PBT in children and adolescents in Kuwait and documenting tumor distribution according to age, sex, histological type and anatomical location. It covered a 17-year period (1995–2011) and was based on the records of the Department of Pathology, Al-Sabah Hospital, Kuwait. During this period all brain tumors in children and adolescents in Kuwait were biopsied in the Department of Neurosurgery, Ibn Sina Hospital, and examined at the Department of Pathology, Al-Sabah Hospital. Despite the fact that this is a hospital -based, rather than population-based study, the results are expected to, more or less, reflect a fair image of the epidemiology of PBT in childhood and adolescence in Kuwait and will serve as a foundation for future studies and planning.

## Materials and methods

Patients below the age of 20 years, at time of diagnosis, were classified as children (0–14 years) and adolescents (15–19 years). Children were further stratified into 5-year age groups- 0–4, 5–9 and 10–14 years. All new cases of primary brain tumors with histological diagnosis in patients within these age groups were identified from the 1995–2011 records of the Department of Pathology, Al-Sabah Hospital Kuwait. Patient’s characteristics, tumor type and anatomical location were analyzed. For comparative analysis, the number of all PBT and high grade tumors diagnosed in adults (ages ≥20 years) during the same period were also extracted.

Histological classification of tumors was based on the “The World Health Organization (WHO) Classification of Tumors of the Central Nervous System (2007)” (Louis et al. 2007). As a result of expected small sample size, clinical classification was used for statistical analysis. Subsequently, Grades I and II tumors were classified as low grade and grades III and IV tumors as high grade.

The mid-year population statistics from 1995–2011 was used in the calculation of crude incidence rates (Population statistics 1995– 2011). Incidence rates were standardized for tumors with a minimum count of 20 on the basis of Segi’s World Population (Segi 1960). Crude age-specific incidence rates for each sex and boys/girls rates ratio (RR) were calculated for PBT in general and selected tumors.

### Definition of terms

Frontal, parietal, temporal and occipital lobes (ICDO site code-C71.1–71-4) were collectively referred to as Lobes.

Ventricular tumors encompass tumors that are completely or largely (≥ two-thirds) within the ventricular system (ICDO site code C71.5).

Pituitary tumor is a collective name for pituitary adenoma and craniopharyngioma (ICDO-site code C75.1–75.2).

Gliomas refers to tumors of presumed glial origin These include astrocytic tumors, ependymoma, oligodendroglioma, mixed glioma and malignant glioma (NOS) and fall under the ICD-O-3 histology codes 9380–9384, 9391–9460, and 9480.

Embryonal tumor is the collective name for medulloblastoma, supratentorial primitive neuroectodermal tumor (sPNET) and atypical teratoid-rhabdoid tumor (AT/RT).

Congenital tumor refers to any tumor that manifests within 60 days of life as defined by Arnstein et al. ( 1951).

## Results

**Figure 1:**
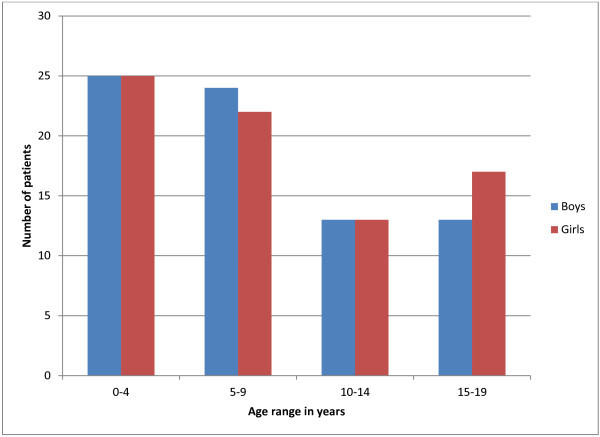
**Age and sex distribution of primary brain tumors before the age of 20 years.**

Between 1995 and 2011, 152 new patients, made up of 75 (49%) boys and 77 (51%) girls, had histologically confirmed PBT in Kuwait. Their ages varied from 24 days to 19 years. Three patients (2%) were less than 2 months old at the time of diagnosis. There were 122 children −62 boys (50.8%) and 60 girls (49.2%) and 30 adolescents −13 boys (43.33%) and 17 girls (56.67%). The general age distribution was as follows: 0–4 years: 50 (32.89%), 5–9 years: 46 (30.26%), 10–14 years: 26 (17.11%) and 15–19 years: 30 (19.74%). The age and sex distribution of PBT in childhood and adolescence is depicted in Figure 1.

### Incidence rates

**Figure 2:**
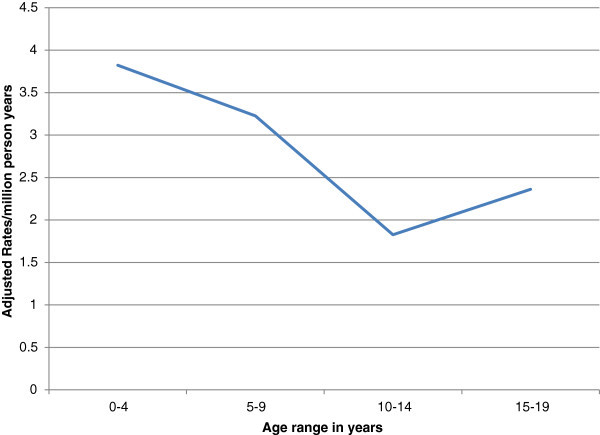
**Distribution of adjusted age-specific incidence rates for primary brain tumors before the age of 20 years.**

**Figure 3:**
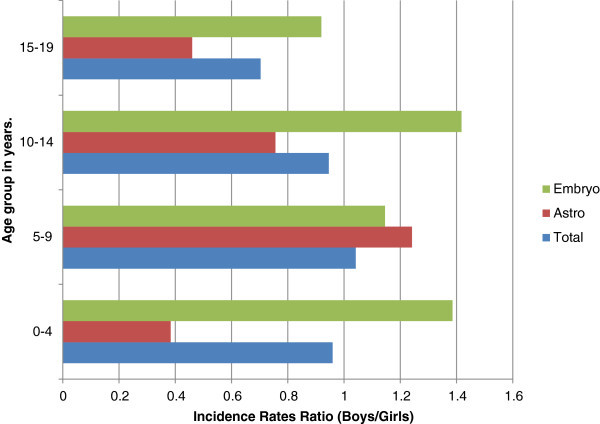
**Incidence rates ratio (boys/girls) for all brain tumors, astrocytic tumors and embryonal tumors.** Astro = Astrocytic; Embryo = Embryonal.

The age-adjusted incidence rate for PBT below the age of 20 years was 11.2/million person-years. The distribution of the adjusted age-specific rates is shown in Figure 2. In all age groups, except 5–9 years, boys had slightly lower age-specific rates than girls (Figure 3).

### Tumor grades

Among 841 adult patients (aged ≥20 years) with PBT, 236 (28%) had high grade tumors. In childhood and adolescence 64 (42%) patients had high grade tumors. Further analysis showed that high grade tumors comprised 47% of childhood and 23% of adolescence tumor. The difference was statistically significant (z = 2.324; p = 0.02). High grade tumors displayed a peak frequency (42%) in the 0–4 years age group and a highly significant inverse relationship with age (r = −0.982; p = 0.006). They occurred in 37 boys (58%) and 27 girls (42%). On the other hand, low grade tumors had a peak frequency (30%) in the age range of 5–9 years and an insignificant inverse relationship with age (r = −0.304; p = 0.696). They were seen in 38 boys (43%) and 50 girls (57%).

### Histological types

The group of tumors known as gliomas constituted 54.1% and 43.3% of all childhood and adolescent tumors respectively. In addition, they formed 25% of childhood and 57% of adolescence high grade tumors.

They comprised astrocytic tumors (67), ependymoma (11) and oligodendroglioma (1).

### Astrocytic tumors

**Figure 4:**
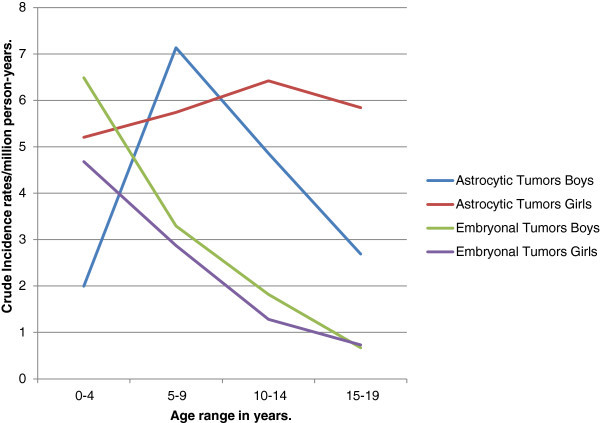
**Crude age-specific incidence rates of astrocytic and embryonal tumors for boys and girls.**

Astrocytic tumors formed 44.08% of all PBT and were diagnosed in 29 boys and 38 girls. Patients’ ages ranged from 1–19 years with an average of 9 years. Most (82.09%) occurred in childhood. The peak frequency (34%) was in the 5–9 years age range. The crude incidence rate was 4.9/million person-years for boys and 5.8/million person-years for girls. The crude age-specific incidence rates peaked between 5 and 9 years for boys and between 10 and 14 years for girls. In general, girls had higher rates in all age groups except 5–9 years (Figure 4). The incidence rates for girls were at least twice as high as those of boys in the 0–4 and 15–19 years age groups (Figure 3).

Astrocytoma was the most common tumor and represented 34.87% of all PBT. In addition, it was the leading tumor in all age groups except 0–4 years. It accounted for about 58% of all tumors seen in the 10–14 years group.

### Low grade astrocytic tumors

Thirty girls and 21 boys had low grade astrocytic tumors. There were 44 cases of pilocytic astrocytoma, 4 cases of diffuse astrocytoma (WHO Grade II) and 3 cases of subependymal giant cell astrocytoma. Pilocytic astrocytoma made up about 29% of PBT and was diagnosed in 19 boys and 25 girls. About 64% of patients with pilocytic astrocytoma were less than 10 years old, while 75% of the tumor was infra-tentorial. One patient with pilocytic astrocytoma had neurofibromatosis. Another patient with subependymal giant cell astrocytoma had tuberous sclerosis.

### High grade astrocytic tumors

Sixteen patients- 8 boys and 8 girls- had high grade astrocytic tumors. These comprised 13 glioblastoma (GBM) and 1 each of anaplastic astrocytoma, malignant pilocytic astrocytoma and high grade astrocytic tumor, not otherwise specified. The small size of the biopsy did not permit further classification of the last case. GBM was the second most common high grade tumor. It accounted for 20% of all high grade tumors but 8.55% of all PBT. All were supratentorial. Its age distribution was bimodal with peaks in 5–9 years (38%) and 15–19 years (31%). One patient (8%) was less than 5 years old.

### Ependymoma

Ependymoma contributed 7.23% to all PBT and occurred in 5 boys and 6 girls aged between 1.5 to 19 years. The median age was 5 years. About 91% of the patients were less than 10 years old. The tumor was infra-tentorial in 8 patients and supra-tentorial in 3. There were 9 WHO Grade II and 2 Grade III tumors.

### Embryonal tumor

Embryonal tumor was the second most common PBT (26.32%), the leading high grade tumor (63%) and the most common tumor (44%) in the age group of 0–4 years. It comprised medulloblastoma (33 cases), supratentorial primitive neuroectodermal tumor (5 cases) and atypical teratoid-rhabdoid tumor (2 cases). Embryonal tumor was diagnosed in 23 boys and 17 girls, aged between 24 days and 19 years. The average age was 5.7 years. About 83% of the patients were younger than 10 years while 55% were less than 5 years old.

It had a crude incidence rate of 3/million person-years. Boys had higher age-specific rates than girls in childhood. The rates ratio varied from 1.14 to 1.41 (Figures 3 and 4).

One patient with medulloblastoma had Kenny Caffey syndrome.

### Pituitary tumor

Sixteen patients (7 children and 9 adolescents) had pituitary tumor. It constituted 30% of adolescence, but only 5.74% of childhood, PBT. There were 9 cases of craniopharyngioma and 7 pituitary adenomas.

Craniopharyngioma affected 5 boys and 4 girls aged 4–15 years with a median age of 9 years. Seven occurred in childhood and 2 in adolescence.

Pituitary adenoma was seen in 6 girls and 1 boy, aged between 16 and 19 years. The median age was 19 years. It formed 78% of all sellar and supra-sellar tumors in adolescence. Six tumors were functional and comprised lactotroph (3), somatotroph (2) and corticotroph (1).

### Choroid plexus tumors

Three boys and 2 girls, aged between 2 months and 2 years had choroid plexus tumors. Three were less than 6 months old.

### Other tumors

These included meningioma (4), neurocytoma (3), germ cell tumor (2) and 1 each of pineoblastoma, chordoma, and lipoma. One patient with meningioma had received radiotherapy for an unspecified cerebellar tumor 15 years earlier.

**Figure 5:**
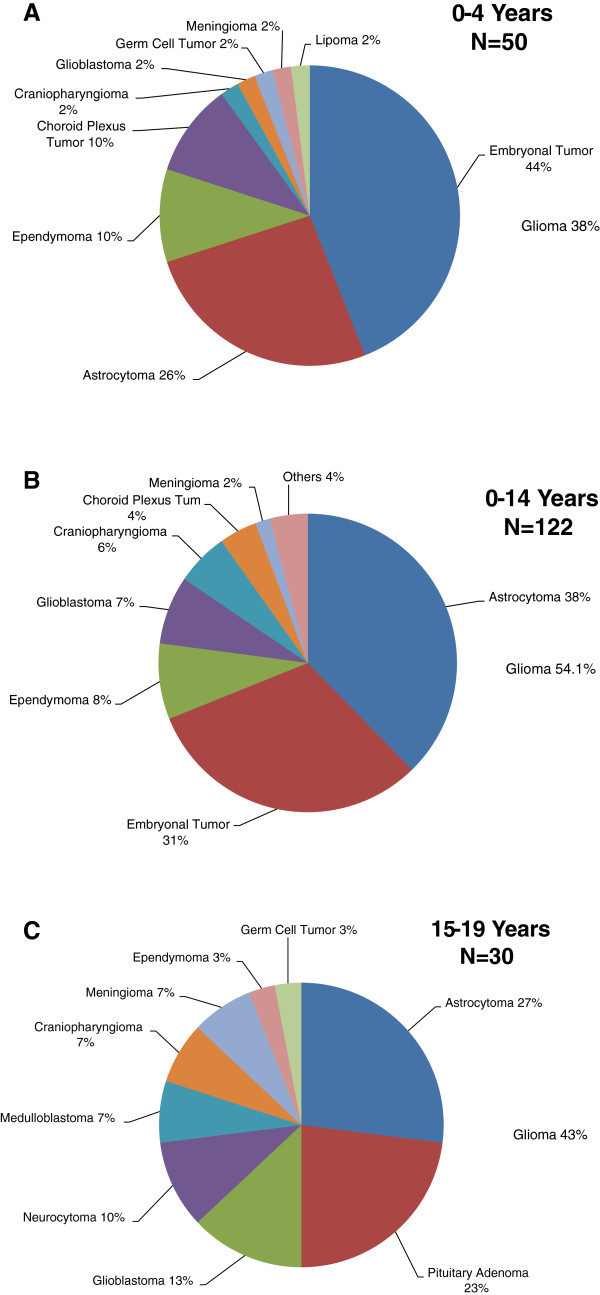
**Distribution of the various histological types of tumors in childhood and adolescence.**

The distribution of the various tumors in childhood and in adolescence is shown in Figure 5. The two most common tumors for the various age groups are presented in Table 1.

**Table 1:** **Most common tumors in the different age groups**

Age range in years	Most common tumor	Second most common tumor
0–4	Embryonal Tumor (44%)	Astrocytoma (26%)
5–9	Astrocytoma (37%)	Embryonal Tumor (24%)
10–14	Astrocytoma (58%)	Embryonal Tumor (19%)
15–19	Astrocytoma (27%)	Pituitary Adenoma (23%)

### Anatomical location

**Figure 6:**
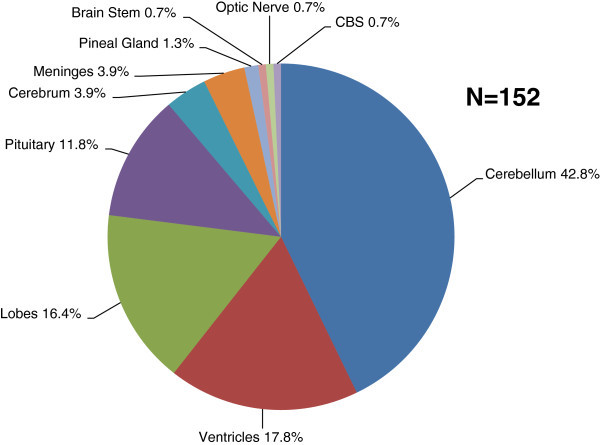
**Distribution of primary brain tumors according to anatomical location.** *CBS = Cerebellum and Brain Stem.

The distribution of childhood and adolescence tumors as a group according to anatomical location is shown in Figure 6. One large tumor involving both cerebellum and brain stem was classified independently. Further analysis reveals differences in pattern between childhood and adolescence tumors as separate entities. In childhood, the most common sites were cerebellum (47%), ventricles (19%) and lobes (17%). In adolescence, they are pituitary (30%), cerebellum (27%) and 13% each for ventricles and lobes. About 46% of cerebellar and intraventricular tumors were high grade.

There were 78 infratentorial and 74 supratentorial tumors.

### Infratentorial tumors

Infratentorial tumors formed 57% of childhood tumors and 27% of tumors in adolescence. They were evenly distributed among both sexes. Their frequency peaked (41%) before the age of 5 years and rapidly declined. The inverse relationship with age was statistically significant (r = −0.964; p = 0.04). About 76% of patients with infra-tentorial tumors were less than 10 years old, while 10% were adolescents. Approximately, 47% of these tumors were high grade.

### Supratentorial tumors

Supratentorial tumors constituted 73% of tumors in adolescence and 43% of childhood tumors. Thirty-six boys and 38 girls had supratentorial tumors. The age distribution was bimodal with peaks in the 5–9 years age group (26%) and 15–19 years (30%). A dichotomy was observed in the age distribution of low and high grade tumors. The low grade tumors peaked at 5–9 (23%) and 15–19 years (36%). Conversely, the high grade tumors had a unimodal distribution with peak (33%) before the age of 5 years.

## Discussion

Despite an inherent selection bias, the results of this study suggest that the epidemiology of primary brain tumors (PBT) in Kuwait, before the age of 20 years, is characterized by low incidence rate and differences in gender preference, distribution of specific histology, topographic dominance and proportion of high grade tumors between childhood and adolescence tumors.

The low incidence rate observed in this study appears paradoxical because of the previously reported relatively high frequency of PBT in this age group. Notwithstanding this oddity, the finding is consistent with reports from other economically developing countries. Stiller and Nectoux have suggested that underdiagnosis may be a major contributory factor to this observation (Stiller & Nectoux 1994). This factor is considered insignificant in Kuwait which has modern diagnostic facilities and easily accessible healthcare delivery system.

A slight male preponderance, with a boys/girls (B/G) ratio of 1.03, is seen in childhood PBT in Kuwait. This is most likely a reflection of the gender distribution in the population at risk (B/G ratio: 1.05). The B/G ratio of PBT in childhood as reported in English Literature varies from 1.08 to 2.52, with Pakistan recording the highest ratio (Kadri et al. 2005; Makino et al. 2010; Mehrazin & Yavari 2007; Farinotti et al. 1998; Ahmed et al. 2007). On the other hand, there is a slight preponderance of female patients in adolescence PBT (B/G ratio: 0.76). Although this is at variance with the sex distribution in the population at risk (B/G ratio: 1.09) it is statistically insignificant (p = 0.17).

Astrocytoma is the most common PBT in childhood and adolescence in Kuwait. The dominance of astrocytoma in childhood is consistent with reports from most countries, including Iran, another Middle-Eastern country (Mehrazin & Yavari 2007; Rickert & Paulus 2001). In contrast, embryonal tumor (medulloblastoma) is the most common childhood PBT in Syria (Kadri et al. 2005). In addition Syria has a significantly higher proportion of craniopharyngioma than Kuwait and Iran. Both observations suggest intra-regional differences in the epidemiology of childhood PBT.

There is a striking difference in topographical distribution of tumors in childhood and in adolescence in this series. Cerebellar tumors are more frequent in childhood than in adolescence, while the converse holds true for pituitary tumors. It is pertinent to note that while no case of pituitary adenoma has been recorded in childhood, it constitutes 80% of sellar tumors in adolescence. Consistent with other reports, it is predominantly functional and has a female predilection (Webb & Prayson 2008).

Comparative analysis indicates that cerebellar and intraventricular tumors are roughly thrice as common in Kuwait as in the United States of America (USA). On the contrary, USA has a higher incidence of brain stem tumors (11%) than Kuwait (1%) (CBTRUS Statistical Report 2012).

Statistically, there is no significant difference in the relative frequencies of infra- and supra-tentorial tumors in the first two decades of life in Kuwait. However, with childhood and adolescence as independent factors, glaring differences appear. Infratentorial tumors dominate in childhood, while adolescence tumors are predominantly supratentorial. Besides, high grade tumors are significantly more frequent in childhood than in adolescence. These differences may be attributed to the influence of embryonal tumors and pilocytic astrocytoma. About 63% of high grade tumors are embryonal. Besides, together with pilocytic astrocytoma, embryonal tumors constitute more than half the total number of tumors seen before the age of 20 years. Both tumors have a proclivity for childhood and infratentorial location.

In Kuwait, similarities in the epidemiology of PBT in adolescence and in adulthood are underscored by predominance of supratentorial tumors, more or less similar frequencies of high grade and pituitary tumors, and rarity of ependymal tumors. Nonetheless, there are contrasts in the distribution of the specific histological types. This is manifested partly by the leading role of pilocytic astrocytoma and meningioma in adolescence and in adulthood respectively and partly by marked differences in the percentage of embryonal tumors and nerve sheath tumors in each group. Nerve sheath tumor was conspicuously absent in this series (Katchy et al. 2011). These discrepancies lend credence to the assertion by Arora et al. that the epidemiology of PBT in adolescence is dissimilar from that of adults (Arora et al. 2009). The unique characteristics of PBT in adolescence are most likely a reflection of the role of adolescence as a transition between childhood and adulthood. Consequently, predominantly childhood tumors tend to be more common in adolescence than in adulthood. Kieran et al. have suggested that a relationship may exist between the different processes of brain development in childhood, adolescence and adulthood and frequency and type of tumors (Kieran et al. 2010).

The over-all pattern of childhood and adolescent PBT in Kuwait is closer to that in Western than in Far Eastern Countries. The relative frequency of germ cell tumors, craniopharyngioma and embryonal tumors support this assertion (Makino et al. 2010; Rickert & Paulus 2001; CBTRUS Statistical Report 2012; Lee et al. 2010).

In general, the frequency of gliomas in childhood and in adolescence is more or less the same in Kuwait and in USA. However, USA has a much higher rate of malignant gliomas than Kuwait. Thus, malignat gliomas form 71% and 74% of high grade tumors in childhood and adolescence respectively in USA (CBTRUS Statistical Report 2012), but 25% and 57% respectively in Kuwait.

Early childhood (0–4 years) appears remarkable in the epidemiology of PBT in Kuwait for several reasons. Firstly, it has the highest age-specific incidence rate and frequency of tumors. Secondly, astrocytic tumors have their lowest boys/girls rates ratio in this age group. It is noteworthy that their rate in girls in this age group is about two and a half times that of boys. Thirdly, the distribution of tumors contrasts sharply with observations in Western countries. Embryonal tumors and gliomas represent about 44% and 38% respectively of all tumors in this age group as opposed to 18% and 52% in USA.

Similarly, the leading tumors in this age group in Germany are astrocytoma and ependymoma (Rickert et al. 1997). Thus, it can be inferred that Kuwait probably has a higher frequency of embryonal tumors in early childhood than Western countries.

Although one patient with Kenny Caffey syndrome had medulloblastoma, the significance of this hereditary syndrome in the etiology of brain tumors has not been established. Nonetheless, 2% of the patients in this series had generally accepted risk factors for PBT. Since hereditary factors were identified in only 1.3% of the cases, they may be considered insignificant etiological factors for childhood PBT in Kuwait.

Finally, about 2% of tumors in this study are congenital. This further confirms the rarity of congenital brain tumors, whose frequency has been reported to vary from 0.5% to 4% among pediatric brain tumors (Manoranjan & Provias 2011).

## Conclusion

The results of this study do not explain the relatively high proportion of primary brain tumors before the age of 20 years in Kuwait. However, they have highlighted the prominent features of childhood and adolescence PBT in Kuwait. Compared with Western countries, Kuwait has a lower incidence of malignant gliomas, but a higher frequency of cerebellar and intraventricular tumors.

The similarity between the pattern of childhood tumors in Kuwait and Iran on one hand and dissimilarity with Syria on the other hand suggests intraregional differences in the Middle-East. Adolescence PBT has distinct epidemiological features different from both childhood and adult PBT.
